# Association between Hypodontia and Angle's Malocclusions among Orthodontic Patients in Kathmandu, Nepal

**DOI:** 10.1155/2022/9595920

**Published:** 2022-12-05

**Authors:** Sanjay Prasad Gupta, Samarika Dahal, Khushboo Goel, Amar Bhochhibhoya, Shristi Rauniyar

**Affiliations:** ^1^Department of Orthodontics and Dentofacial Orthopedics, Tribhuvan University Teaching Hospital, MMC, Institute of Medicine, Tribhuvan University, Kathmandu, Nepal; ^2^Department of Oral Pathology and Forensic Dentistry, Tribhuvan University Teaching Hospital, MMC, Institute of Medicine, Tribhuvan University, Kathmandu, Nepal; ^3^Department of Periodontics, Tribhuvan University Teaching Hospital, MMC, Institute of Medicine, Tribhuvan University, Kathmandu, Nepal; ^4^Department of Prosthodontics, Tribhuvan University Teaching Hospital, MMC, Institute of Medicine, Tribhuvan University, Kathmandu, Nepal; ^5^Dental Villa-Orthodontic Center and Speciality Dental Clinic, Kathmandu, Nepal

## Abstract

**Background:**

Disturbances during the early tooth development stages may result in the congenital absence of teeth. The purpose of this study was to assess the relationship between hypodontia and Angle's malocclusions.

**Materials and Methods:**

The sample comprised 601 orthodontic patients' pretreatment records (242 men and 259 women), selected from the achieved orthodontic records. Developmental anomalies of teeth affecting the number were examined on dental panoramic radiographs. Based on Angle's classification, pretreatment dental casts were assessed and classified into different classes of malocclusion. The relationship between hypodontia and different classes of malocclusion was evaluated using the chi-square test.

**Results:**

The prevalence of tooth agenesis was 7.48%, that is, 45 out of 601 samples. There were a total of 72 (0.42%) missing teeth, excluding the third molars. The most frequent missing tooth was the maxillary lateral incisor (35, 48.61%), followed by the mandibular lateral incisor (14, 19.44%), the mandibular central incisor (6, 8.33%), the mandibular second premolar (5, 6.294%), and the maxillary second premolar (4, 5.55%). Hypodontia was more common in the upper jaw. Although hypodontia was mostly seen in Class I malocclusion patients (7.87%), followed by Class II malocclusion patients (6.99%) and least in Class III malocclusion patients. However, there was no significant difference in hypodontia among different classes of malocclusions (*p* = 0.352).

**Conclusion:**

The most frequently missing tooth was the maxillary lateral incisor, followed by lateral and central mandibular incisors and mandibular second premolars, while excluding the third molars. The present study did not find any association between various types of malocclusions and hypodontia.

## 1. Introduction

Hypodontia is a condition in which the number of teeth in the jaws is reduced due to developmental disturbances of the teeth. The prevalence of hypodontia varies considerably according to ethnic group, gender, and geographical location [1–3]. Hypodontia is more common in some populations than others, with a range of 2.2 to 6.3% [2–4]. In permanent dentition, females have a higher ratio than males, with a ratio of 1.37 : 1[3]. Hypodontia is influenced by both hereditary and environmental factors and it is more prevalent in the maxilla than in the mandible [2].

There are three types of tooth agenesis: hypodontia, oligodontia, and anodontia. Oligodontia is a severe type of hypodontia characterized by an absence of more than six permanent teeth, excluding the third molars, whereas anodontia refers to the complete absence of teeth [5].

Patients with hypodontia may suffer from an unfavorable esthetic appearance, inarticulate pronunciation and reduced chewing ability that ultimately affect their communication behavior, self-esteem, and professional performance [6, 7].

Hypodontia can be syndromic or nonsyndromic. Complex developmental syndromes linked to a congenitally missing tooth or teeth are referred to as “syndromic tooth agenesis” [8]. A congenitally absent tooth in an isolated form that is not linked to any other significant birth defects is referred to as nonsyndromic tooth agenesis [9].

Hypodontia patients may present with some skeletal features such as a short and retrognathic maxilla, prognathic mandible, and shorter lower anterior facial height [10].

Numerous studies have associated hypodontia with various developmental anomalies such as taurodontism, impacted canines, peg-shaped lateral incisors, and developmental enamel defects [11, 12]. Impaction, hypodontia, and microdontia were the most common developmental anomalies seen in orthodontic patients [13–15]. In deciduous dentition, tooth agenesis have increased the risk of infraocclusion of molars and gemination or fusion of incisors [16]. Study showed an association between dental anomalies and Class II, division 2 malocclusion which indicates a strong genetic influence for the development of malocclusion [17, 18]. On the other hand, Class III and Class II, division 1 malocclusions have a pattern of anomalies similar to that of the general population [19].

Congenitally missing teeth can affect the occlusal and molar relationships of the upper and lower jaws. Class II and Class III malocclusion when associated with hypodontia will create a great challenge in managing the case, hence requiring a thorough knowledge and experience in order to provide better facial aesthetics. However, the correlation of hypodontia to different categories of malocclusion is very poorly researched. The present study examined the association between hypodontia and Angle's classes of malocclusion among orthodontic patients in Kathmandu, Nepal. This study will be carried out to fill this gap, and it is believed that the findings will aid further investigation.

## 2. Materials and Methods

The study sample comprised archived pretreatment records of six hundred one orthodontic patients who came for orthodontic treatment at Tribhuvan University Teaching Hospital and Dental Villa-Orthodontic Center and Specialty Dental Clinic, Kathmandu, Nepal. The sample size for this study was calculated by using the formula *n* = *Z*^2^*pq*/*d*^2^, where *Z* = 1.96, value of *p* is taken as 0.5, *q* = 1 − *p* = 0.5, allowable error (*d*) = 0.04 (96%), and *n* is required sample size. Based on these parameters, the required sample size was 600.25. Hence, a total of 601 patients were selected.

The orthodontic records of the patients between 10 and 35 years of age who came for orthodontic treatment with good-quality panoramic radiographs and casts were included in this study.

Patients with a history of orthodontic treatment, systemic disease, or craniofacial anomalies/congenital syndromes, missing teeth due to tooth decay, avulsions, or extractions during dental therapy, were excluded from this study. Before conducting the study, ethical approval was obtained from the institutional review committee of the Institute of Medicine [Ref.13 (6–11) E2,079/080].

### 2.1. Determination of Hypodontia

Dental panoramic radiographs were evaluated by a single operator. A tooth was identified as congenitally missing only if the mineralization of its crown could not be identified on an orthopantomogram. The evaluation of the digital orthopantomogram was done on a computer screen with a resolution of 1280 × 800 pixels (*MacBook Air, Apple computer, California, USA).* Gender, patient age at the time of radiography, number of missing teeth, and location of missing teeth were recorded and entered into an Excel sheet.

### 2.2. Determination of Angle's Malocclusion

All dental casts were evaluated for occlusal relationships by a single operator and classified into three classes of malocclusion based on Angle's classification: Class I malocclusion, Class II malocclusion, and Class III malocclusion [20].

### 2.3. Statistical Analysis

The data were collected and transferred to an MS Excel sheet. The dataset was verified and statistically analyzed using Statistical Package for the Social Sciences (SPSS) Statistics, version 21.0 (IBM Corp., Armonk, N. Y., USA) with a confidence level set at 95% (*P* < 0.05) to test for significance. Descriptive statistics were used to analyze dental agenesis and classify malocclusions among orthodontic patients. After two weeks, one hundred twenty-five orthopantomograms were randomly chosen for the Kappa test (0.91) to assess intraobserver reliability.

The association between hypodontia and different classes of malocclusion was assessed using Pearson's chi-square test.

## 3. Results

**Figure 1:**
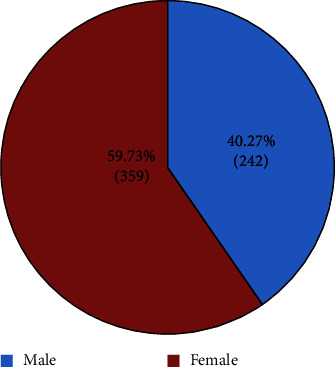
Gender distribution of the samples.

The study sample of 601 patients was comprised of 242 (40.27%) males and 359 (59.73%) females (Figure 1). The mean age of the patients was 16.42 ± 3.428 years (Male: 17.31 ± 3.11 years; Female: 16.15 ± 4.46). The distribution of missing teeth according to the type of tooth and location is depicted in Table 1.

**Figure 2:**
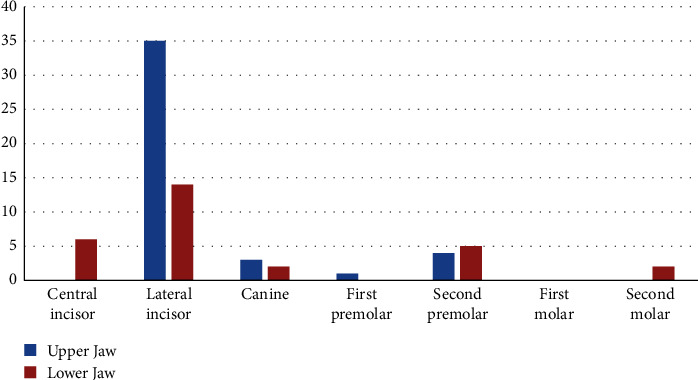
Distribution of the number of missing teeth in the upper and lower jaw.

The prevalence of dental agenesis was 7.48% (45 out of 601 samples). The total number of missing teeth was 72 (0.42%), excluding the third molars. Agenesis was more prevalent in the upper jaw (43, 60%) compared to the lower jaw (29, 40%). The most common missing teeth were maxillary lateral incisors (35, 48.61%), followed by mandibular lateral incisors (14, 19.44%), mandibular central incisors (6, 8.33%), mandibular second premolars (5, 6.94%), and maxillary second premolars (4, 5.55%), excluding the third molars (Table 1, Figure 2).

The total sample of 601 subjects was distributed into three groups based on Angle's classification of malocclusion. Patients with Class I malocclusion constituted about 65.55% (394), Class II malocclusion about 30.94% (186), and Class III about 3.49% (21) of the samples. Hypodontia was seen mostly in Class I (31, 7.87%), followed by Class II (13, 6.99%) and least in Class III (1, 4.77%) malocclusion patients. However, the chi-square test did not have a significant difference (*p* = 0.352) in hypodontia among different classes of malocclusions (Table 2).

## 4. Discussion

Tooth agenesis occurs due to a defect in the early stages of tooth development, which can be confirmed by clinical and radiographic examination. A tooth that has not emerged in the oral cavity and whose dental crypt is not discernible on a radiograph confirms agenesis. [21] Agenesis results in hypodontia.

The management of patient with hypodontia is either treated with orthodontic space closure, replaced with a prosthesis or both, so it requires a multidisciplinary approach that involves specialists from different dental specialties. Prosthetic replacement with endosseous implants needs various orthodontic considerations such as uprighting mechanics, extrusion/intrusion, delayed space opening, and orthodontic implant site-switching to create, preserve, or augment the implant site [10].

The prevalence of hypodontia in the present study was 7.48%, excluding the third molar. Previous studies have reported the lowest prevalence of hypodontia to be 2.8% in Malaysia [22] and the highest to be 12.6% in Germany [23].

Chung et al. [24] also estimated a higher prevalence of 11.2% in the Korean population and Polder et al. [3] showed 10.1% in the Norwegian population. [3] These wide ranges of prevalence values indicated that racial, geographic, gender, and genetic differences, as well as the huge differences in the sample size and criteria of selection played a fundamental role in the varied results of studies of hypodontia. Hence, comparison of the result of this study is very limiting compared to other previous studies.

The prevalence of oligodontia in this study was 0.33%; that is, only 2 individuals showed more than 6 missing teeth which is similar to the study by Vahid-Dastjerdi et al. [2] conducted in Iranian population, whereas it is much lower than Peker et al. [25] study (7%) conducted in the Turkish population.

The present study showed a predominance of hypodontia in the maxilla, which is similar to the study by Vahid-Dastjerdi et al. [2] and Ali and Hussain 26, while in contrast to this, other studies showed mandibular predominance [27].

The present study showed the maxillary lateral incisor as the predominant tooth to undergo agenesis which is similar to Vahid-Dastjerdi et al.'s study in the Iranian population [2], Abu [28] and Polastri [29] in the Italian population, and Ali and Hussain [26] in the Pakistani population. Other studies [30–34], on the other hand, found that mandibular second premolars were the most commonly missing teeth, followed by maxillary lateral incisors.

In the present study, hypodontia was seen mostly in class I malocclusion, followed by class II malocclusion, and least in class III malocclusion, without a significant difference between them. This is similar to the study conducted among Brazilians by Pedreira et al. [30] In contrast to this, studies by Stefani et al. [34] and Al-Amiri et al. [35] found a positive correlation between hypodontia and Class II malocclusion. Similarly, Burzynski and Ecobar [27], Vahid-Datjerdi et al. [2]; and Ali and Hussain [26] found a higher frequency of missing teeth in patients with Class III malocclusion.

The limitations of this study are that it was conducted among the orthodontic patients rather than in general populations and ethnicity was not considered in this study.

The findings of the present study may vary among diverse populations and ethnic groups. Thus, multicenter collaborative studies in diverse populations with healthy controls in a larger sample size are recommended for comprehensive assessment. Additionally, future studies can be performed in order to evaluate other variables and their possible relationship with hypodontia, such as sella turcica bridging [36] and different growth and skeletal malocclusion patterns [37].

## 5. Conclusion

The most common missing tooth was the maxillary lateral incisor, followed by the mandibular lateral incisor, mandibular central incisor, and mandibular second premolar, while excluding the third molars. The present study did not show an association between hypodontia and different classes of malocclusion.

## Figures and Tables

**Table 1:** Distribution of missing teeth according to the type of tooth and its location.

Type of tooth	Upper jaw *N* (%)	Lower jaw *N* (%)	Total *N* (%)
Central incisor	0	6 (8.33%)	6
Lateral incisor	35 (48.61%)	14 (19.44)	49
Canine	3 (4.16%)	2 (2.77%)	5
First premolar	1 (1.38%)	0	1
Second premolar	4 (5.55%)	5 (6.94%)	9
First molar	0	0	0
Second molar	0	2 (2.77%)	2
Total	43 (60%)	29 (40%)	72

**Table 2:** Distribution of patients with hypodontia among the three classes of malocclusion.

Malocclusions type	Study sample *N* (%)	Hypodontia		*P*value (chi-square test)
		Yes *N* (%)	No *N* (%)	
Class I	394 (65.55%)	31 (7.87%)	363 (92.13%)	0.352
Class II	186 (30.94%)	13 (6.99%)	173 (93.01%)	
Class III	21 (3.49%)	1 (4.77%)	20 (95.23%)	
Total	601 (100%)	45 (7.49%)	556 (92.51%)	

*p* < 0.05 = Statistically significant.

## Data Availability

Data can be made available upon reasonable request to the author.
